# Hybrid transformer-CNN model for accurate prediction of peptide hemolytic potential

**DOI:** 10.1038/s41598-024-63446-5

**Published:** 2024-06-20

**Authors:** Sultan Almotairi, Elsayed Badr, Ibrahim Abdelbaky, Mohamed Elhakeem, Mustafa Abdul Salam

**Affiliations:** 1 Department of Computer Science, Faculty of College of Computer and Information Sciences, Majmaah University, 11952 Majmaah, Saudi Arabia; 2 Department of Computer Science, Faculty of Computer and Information Systems, Islamic University of Madinah, 42351 Medinah, Saudi Arabia; 3 Scientific Computing Department, Faculty of Computers and Artificial Intelligence, Benha University, Benha, Egypt; 4 The Egyptian School of Data Science (ESDS), Benha, Egypt; 5 Artificial Intelligence Department, Faculty of Computers and Artificial Intelligence, Benha University, Benha, Egypt; 6 Department of Computer Science, College of Arts and Science, Wadi Addawasir, Prince Sattam Bin Abdulaziz University, 16273 Al-Kharj, Saudi Arabia

**Keywords:** Peptides, Hemolysis, Deep learning, Convolutional neural networks (CNNs), Transformers, Drug design, Hemolytic prediction, Biochemistry, Peptides

## Abstract

Hemolysis is a crucial factor in various biomedical and pharmaceutical contexts, driving our interest in developing advanced computational techniques for precise prediction. Our proposed approach takes advantage of the unique capabilities of convolutional neural networks (CNNs) and transformers to detect complex patterns inherent in the data. The integration of CNN and transformers' attention mechanisms allows for the extraction of relevant information, leading to accurate predictions of hemolytic potential. The proposed method was trained on three distinct data sets of peptide sequences known as recurrent neural network-hemolytic (RNN-Hem), Hlppredfuse, and Combined. Our computational results demonstrated the superior efficacy of our models compared to existing methods. The proposed approach demonstrated impressive Matthews correlation coefficients of 0.5962, 0.9111, and 0.7788 respectively, indicating its effectiveness in predicting hemolytic activity. With its potential to guide experimental efforts in peptide design and drug development, this method holds great promise for practical applications. Integrating CNNs and transformers proves to be a powerful tool in the fields of bioinformatics and therapeutic research, highlighting their potential to drive advancement in this area.

## Introduction

In recent years, the prediction of hemolytic activity in peptides has become a critical focus in biomedical and pharmaceutical research^1–3^. Hemolysis, the process involving the rupture of red blood cells, has substantial implications for drug development and therapeutic design^4,5^. This study introduces a sophisticated computational approach employing CNNs and transformers to enhance the precision and efficiency of predicting hemolytic potential in peptides. The background of this investigation is underscored by the intricate nature of the evaluation of hemolytic activity and the constraints associated with conventional experimental approaches. Conventional methodologies often require significant time and resources, provoking a paradigm shift towards computational methods. In this context, advanced deep learning architectures, such as CNNs and transformers, have emerged as promising tools to navigate the complexities inherent in unraveling the sequence-structure relationships governing hemolysis in peptides. The research problem addressed in this study revolves around the imperative to improve the accuracy and efficiency of predicting hemolytic potential. Traditional experimental approaches are not only resource-intensive but also time-consuming. Computational methods provide a viable alternative, and our hybrid architecture uniquely bridges this gap by combining CNN's local pattern detection with transformers global relationship comprehension, resulting in a deeper understanding of hemolytic activity determinants.

The field of predicting hemolytic activity in peptides is fundamental to our study. To better understand this complex area, we delve into previous research using a range of computational methods, carefully examining their strengths and weaknesses. By synthesizing this literature, we provide a valuable framework for our research, shed light on current knowledge gaps, and pave the way for our innovative approach. Past studies relied on feature engineering or shallow models, often overlooking intricate long-range dependencies within peptide sequences^6–11^. Although traditional methods offer valuable information, they have restrictions in terms of scalability, efficiency, and the ability to understand complex sequence-structure relationships. As a result, researchers have increasingly relied on computational methods to enhance and streamline prediction. Numerous computational strategies have been investigated, including machine learning algorithms and advanced deep learning architectures. Machine learning models^12^, including support vector machines (SVM) and random forests, have been applied to predict hemolytic potential based on peptide sequences^12–17^. Deep learning models including RNN and transfer learning models, were used^18,19^. Although these methods have shown considerable predictive abilities, their effectiveness is highly dependent on the specific features chosen and may not fully capture intricate connections within peptide sequences. However, in recent times, deep learning techniques such as CNNs and transformers have emerged as powerful tools for automatically extracting hierarchical characteristics and comprehending long-range relationships in sequences^20^. Using these architectures, we can potentially improve the precision and speed in predicting hemolytic activity. The specialized design of CNNs allows effective detection of local patterns, while the innovative use of attention mechanisms in transformers enables the identification of broader connections within sequences^19,21^. The proposed approach was built on this literature to contribute a novel perspective to predicting hemolytic activity. This synergistic combination enables our model to learn complex sequence-structure relationships with exceptional accuracy, exceeding the limitations of previous methods. The critical insights drawn from existing literature guide our methodology, laying the groundwork for a comprehensive and innovative approach to predicting hemolytic activity in peptides. Critically, theoretical modeling approaches based on ordinary differential equations (ODEs) have been instrumental in predicting diseases and deciphering intricate biological processes. Studies utilizing ODE-based theoretical modeling, such as those referenced^22–24^ provide valuable insights into dynamic systems and can complement our computational framework for predicting hemolytic activity. By incorporating these theoretical modeling paradigms into our discussion, we aim to not only enhance the depth of our analysis but also highlight future research directions. The integration of computational methods with theoretical modeling promises to further advance our understanding of hemolysis in peptides, ultimately contributing to more effective drug design and therapeutic strategies. For more details on the identification of peptides using mathematical models, the reader can refer to DiMaggio et al.^25^. On the other hand, for more details on how to formulate real-world problems as mathematical models, the reader is referred to Badr et al.^26–28^.

The advancement of interaction prediction research in computational biology, particularly the use of graph neural networks (GNNs) for miRNA-lncRNA interaction prediction, has provided valuable insights into genetic markers and non-coding RNAs. It is essential to cite pivotal computational models in this domain, such as those detailed in studies^29–36^, which have contributed significantly to the field. Furthermore, acknowledging the progress in interaction prediction research across various computational biology domains is vital. These studies offer valuable insights into genetic markers and associated diseases, underscoring the importance of referencing key computational models within these domains. Relevant studies^37,38^ should be included to highlight the advancements and contributions to the field.

In this paper, our structure is as follows: after this introduction, we will dive into the methodology we utilized to construct and train our predictive models, exploring the reasoning behind incorporating CNNs and transformers. We will then discuss our results and evaluate their performance. Finally, we emphasize the importance of our research and suggest potential avenues for further advancement in predictive modeling for peptide design and biomedical applications.

## Data and methods

In this section, we provide a detailed overview of the datasets used and the methodology used in our study to predict hemolytic activity in peptides utilizing CNNs and transformers.

### Data collection

Our research uses a variety of datasets, ensuring that our predictive models are accessible and widely applicable. The main datasets utilized in this investigation comprise RNN-Hem^18^, Hlppredfuse^12^, and Combined^19^. These datasets incorporate a diverse set of peptide sequences with documented hemolytic activities that serve as the basis for the development, validation, and testing phases of our models.

Table 1 presents a comprehensive overview of the datasets utilized in our research, emphasizing their distinct sources and composition of positive (hemolytic) and negative sets (non-hemolytic). The datasets, namely RNN-Hem, Hlppredfuse, and Combined, have been curated from reputable sources in the field. Each dataset contributes to the diversity of our study by incorporating a wide range of peptide sequences with documented hemolytic activities. RNN-Hem Sourced from Capecchi et al.^18^, this dataset includes 1359 instances in the positive set and 1198 instances in the negative set. Hlppredfuse^12^ obtained from Hasan et al.^12^, this data set comprises 1096 instances in the positive set and 2422 instances in the negative set. Combined with an extract from Salem et al.^19^, this data set incorporates 3007 instances in the positive set and 4172 instances in the negative set.

**Table 1:** Overview of data sets used in the study.

Dataset	Source	Positive set	Negative set
RNN-Hem	Capecchi et al.^18^	1359	1198
Hlppredfuse	Hasan et al.^12^	1096	2422
AMP-Combined	Salem et al.^19^	3007	4172

These datasets are crucial for the success of our model development process. By incorporating a variety of sources and a large number of instances, our predictive models can utilize a diverse and comprehensive sample. This improves their strength and ability to be applied in various situations. In the following sections, we will discuss in detail the techniques used in handling and harnessing these datasets for training and assessing our models.

### Data representation

The way we represent peptide sequences profoundly influences the ability of deep-learning models to unlock their hemolytic potential. Automated representation based on deep learning of biological sequences is effective while saving time and effort in traditional methods of gathering information^39^. A thoughtfully designed numerical representation not only captures the essence of each amino acid but also cultivates a structured landscape where patterns of hemolytic activity can emerge. In this pursuit, we embarked on decoding the hidden language of peptides, carefully crafting a representation that enables our models to delve into the depths of peptide sequences and illuminate their hidden relationships with hemolysis. Each peptide sequence was segmented into its fundamental amino acid units, creating a vocabulary of 20 distinct amino acid symbols. Each amino acid token was assigned a unique numerical index, effectively translating the symbolic sequence into a numerical format suitable for computational processing. To maintain consistency in input dimensions for deep learning models, we padded sequences with zeros up to a fixed maximum length of 50. Given that most of the peptides in our datasets possess lengths below 50, we opt for this maximum length to efficiently represent the majority of sequences while maintaining sufficient capacity for potential long-range dependencies within this range. This ensures a uniform input structure, even with varying sequence lengths. Through this carefully designed numerical representation, we transformed the raw peptide sequences into a structured format that empowers our deep learning models to uncover the intricate relationships between amino acid composition and hemolytic potential.

**Figure 1:**
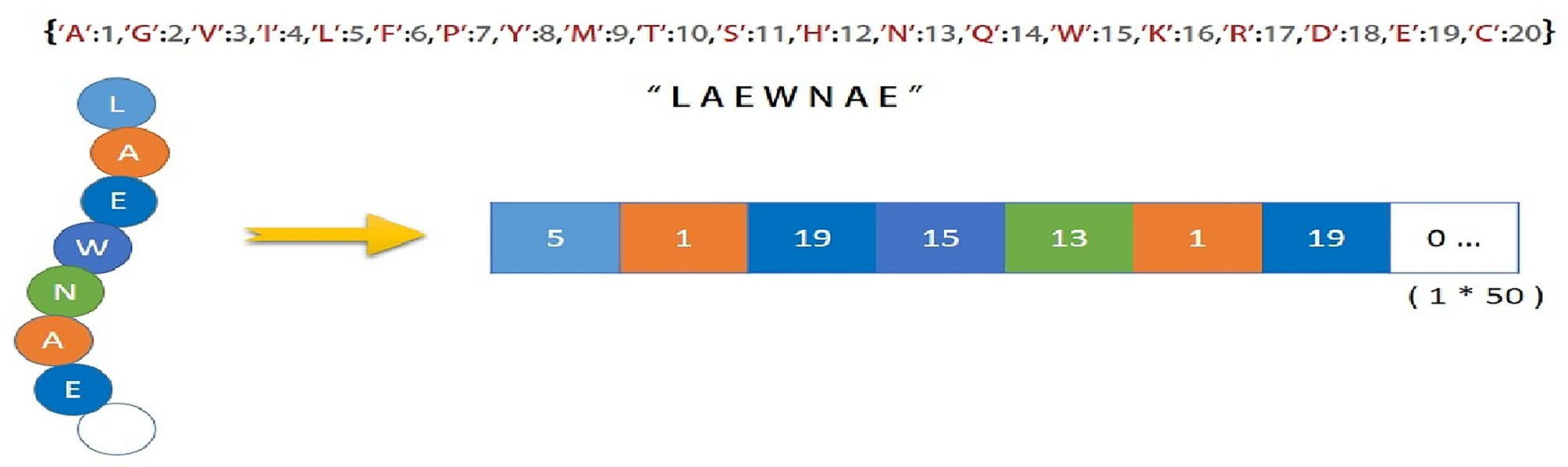
encoding applied to the peptide sequence.

As shown in Fig. 1, each amino acid within the peptide sequence (LAEWNAE) is transformed into a unique numerical index. For example, the first amino acid L is represented as 5. This encoding preserves the distinct identity of each amino acid while facilitating efficient processing by deep learning models. By padding shorter sequences with zeros up to a maximum length of 50 (as shown in the figure), we ensure a consistent input format regardless of the peptide's actual length, enabling the models to focus on the relevant sequence patterns.

### Methodology

**Figure 2:**
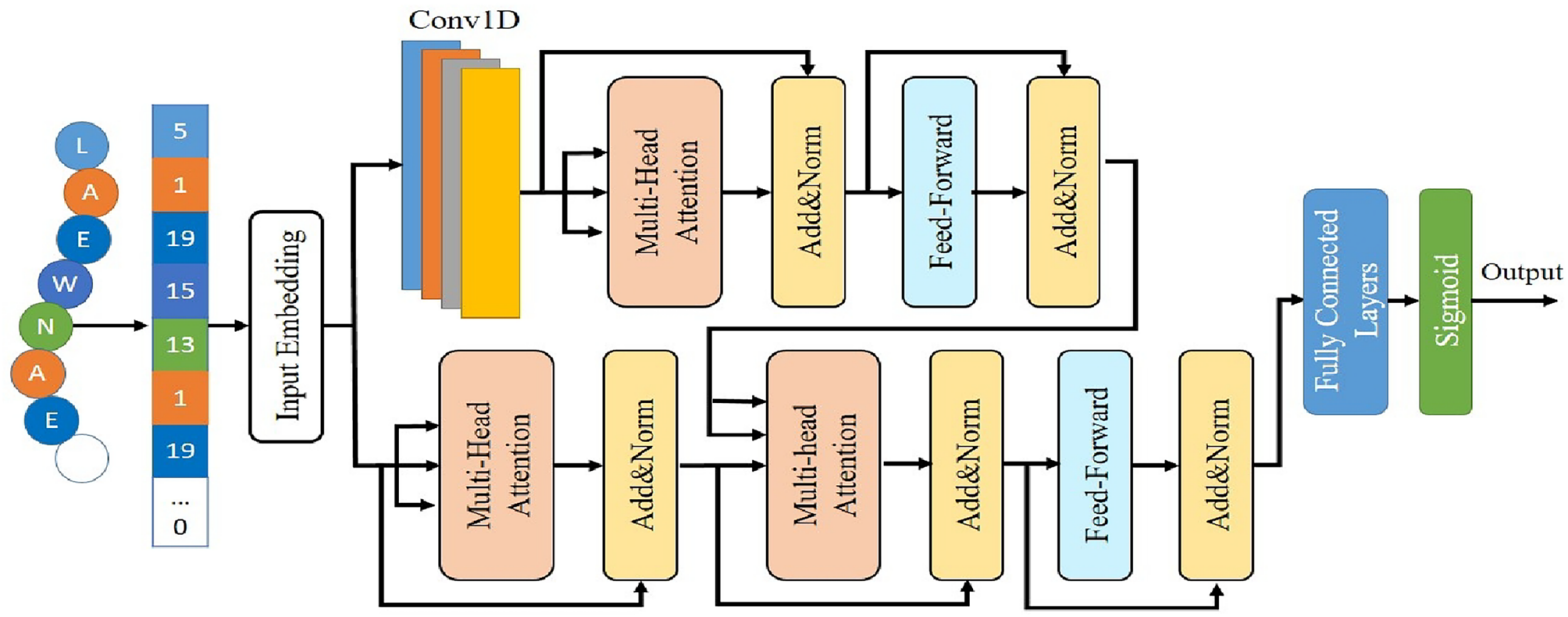
Hybrid transformer-CNN architecture for predicting hemolytic activity of peptides.

The intricacies of peptide hemolysis are analogous to deciphering a complex puzzle, where individual amino acids serve as the pieces and their arrangement dictates the hemolytic potential. In this effort, we constructed a deep learning architecture that seamlessly integrates local and global analyses, as shown in Fig. 2, harnessing the complementary strengths of CNNs and transformer-based attention mechanisms.

At first, CNNs play a pivotal role as they meticulously scan the peptide sequence. They diligently detect recurring patterns, examine the bonds between adjacent amino acids, and unravel the close-range connections that contribute to the fundamental components of hemolytic activity. Similarly to recognizing familiar melodies, CNNs establish a solid understanding of how local collaborations shape the initial characteristics of the hemolytic profile. However, the complexity of the melody goes beyond these immediate harmonies. Here, the Transformers take the lead. With attention mechanisms that span the entire sequence, they carefully study the subtle relationships between distant amino acids. This global perspective unveils long-range collaborations that can enhance or mitigate the hemolytic tendencies established by local motifs. These previously overlooked connections now become integral components, enriching the model's understanding of the peptide's overall hemolytic potential. The synergy between local analysis and global exploration is fundamental to the power of our architecture. The insights obtained, whether short-range motifs identified by CNNs or long-range connections revealed by transformers undergo meticulous processing by dedicated feed forward networks^40^. These processes delicately shape the raw data, providing an all-encompassing description of the subtle mechanisms that drive hemolytic activity. It is like extracting the very essence of a puzzle, capturing every subtle detail and interplay that forms the hemolysis profile.

Throughout the training process, we carefully select a specific set of hyperparameters to enhance the performance of our model. These hyperparameters consist of pre-training adjustments that impact the behavior of the model but are not acquired during the training itself. In this context, some noteworthy examples of hyperparameters include the quantity and dimensions of filters utilized in the convolutional layers, the size of pooling windows employed in the pooling layers, the number of neurons within the fully connected layers, the optimizer's learning rate, the duration of time to train the model, as well as the batch size. For further details, please refer to Table 2 which outlines the specific hyperparameters used during the training process.

**Table 2:** Parameter settings for the proposed model.

Parameters	Value
Number of convolutional layers	1
Number of dense layers (FC)	2
Number of filters	[256]
Filter length	[5]
Hidden neurons	[512, 256]
vocab_size	1024
Activation function (FC)	ReLU
Activation function output	Sigmoid
Batch size	32
Learning rate	0.0001
Optimizer	Adam
Loss function	Binary cross entropy

### Software and hardware

The development and execution of machine learning models were carried out seamlessly using a comprehensive set of software and hardware resources. Python (3.10) emerged as the primary programming language, supported by essential libraries such as Pandas, NumPy, Matplotlib, and scikit-learn for data manipulation, analysis, and visualization. Deep learning models were implemented with TensorFlow (2.13.0). In terms of hardware, Kaggle computational resources, including GPU (GPU T4 ×2) capabilities, were used for model training and evaluation.

### Model evaluation

To evaluate the performance of the hybrid Transformer-CNN model, we used accuracy (Acc), precision, recall, Area under the ROC Curve (ROC-AUC), and Matthews correlation coefficient (MCC)^41^. The evaluation metrics are defined in the following equations:

1 $$\text{Acc}= \frac{TP+TN}{TP+TN+FP+FN} \times 100$$

2 $$\text{Precision}= \frac{TP}{TP+FP} \times 100$$

3 $$Recall= \frac{TP}{TP+FN} \times 100$$

4 $$\text{Mcc}= \frac{TP \times TN - FP \times FN}{\sqrt{(TP + FP)(TP + FN)(TN + FP)(TN + FN)}}$$

## Results

In this section, we present the performance metrics of our proposed hybrid transformer-CNN architecture model across three distinct datasets. RNN-Hem^18^, Hlppredfuse, and Combined. Furthermore, a comprehensive comparative analysis with previously used methods further elucidates the efficacy of our model.

Table 3 shows that the model achieved substantial accuracy (79.69%), precision (82.93%), recall (76.69%), ROC-AUC (0.861), and MCC (0.5962) in the RNN-Hem dataset, indicating its ability to identify hemolytic activity within peptide sequences. Demonstrated exceptional performance with high accuracy (96.16%), precision (93.27%), recall (94.55%), ROC-AUC (0.976), and MCC (0.9111) in the Hlppredfuse^12^ dataset, showcasing the robustness of the model in predicting hemolytic potential. The Combined dataset displayed commendable metrics, with notable accuracy (89.28%), precision (87.59%), recall (86.41%), ROC-AUC (0.942), and MCC (0.7788), highlighting the consistency of the model in various datasets. The hybrid transformer-CNN architecture model consistently exhibits strong predictive capabilities across varied datasets, underscoring its versatility and effectiveness in accurately predicting hemolytic potential in peptides.

**Table 3:** Performance of the proposed model in the three data sets.

Dataset	Accuracy (%)	Precision (%)	Recall (%)	ROC-AUC	MCC
RNN-Hem^18^	79.69	82.93	76.69	0.861	0.59
Hlppredfuse^12^	96.16	93.27	94.55	0.976	0.91
AMP-Combined^19^	89.28	87.59	86.41	0.942	0.77

In Table 4, our proposed hybrid transformer-CNN architecture model exhibited competitive or superior metrics in the RNN-Hem dataset^18^, showcasing its effectiveness in achieving comparable or even better predictive performance compared to established classifiers. AMPDeep^19^ demonstrated competitive accuracy, precision, recall, ROC-AUC, and MCC, positioning itself as a strong contender against traditional classifiers. Existing classifiers, namely SVM-Hem^18^, RF-Hem^18^, and RNN-Hem^18^, achieved moderate performance but were surpassed by the proposed model and AMPDeep^19^. Moving to Table 5, our proposed model outperformed existing classifiers in the Hlppred-Fuse dataset^12^ in terms of accuracy, precision, recall, ROC-AUC, and MCC, highlighting its efficacy in accurately predicting hemolytic potential. Although AMPDeep^19^ showed strong performance metrics, the proposed model surpassed it in multiple evaluation criteria. The existing classifiers exhibited varied performance, underscoring the superiority of our proposed model in predicting hemolytic activity. In Table 6, the proposed model demonstrated better accuracy, precision, recall, ROC-AUC, and MCC compared to existing classifiers in the Combined data set^19^, indicating its robustness in predicting hemolytic potential. Although AMPDeep^19^ showed competitive performance, the proposed model outperformed it in multiple evaluation metrics. This collective evidence underscores the consistent effectiveness of our proposed hybrid transformer-CNN architecture model in predicting hemolytic activity across interdisciplinary datasets, positioning it as a powerful and versatile tool in computational biology.

**Table 4:** Comparison of the proposed model with the previous methods in the RNN-Hem dataset.

Classifier	Accuracy (%)	Precision (%)	Recall (%)	ROC-AUC	MCC
SVM-Hem^18^	73	72	58	0.69	0.44
RF-Hem^18^	77	81	60	0.8	0.53
RNN-Hem^18^	76	70	76	0.87	0.52
AMPDeep^19^	**79.97**	79.88	**83.28**	**0.8723**	**0.5972**
Proposed model	79.69	**82.93**	76.69	0.861	0.5962

Bold values are for the best-performing model.

**Table 5:** Comparison of the proposed model with the previous methods in the HLPpred-Fuse dataset.

Classifier	Accuracy (%)	Precision (%)	Recall (%)	ROC-AUC	MCC
HLPpred-Fuse^12^	–	–	84.5	0.967	0.823
HemoPI^16^	–	–	80.4	0.952	0.754
HemoPred^42^	–	–	65.2		0.34
AMPDeep^19^	93.69	86.67	88.24	0.9716	0.8324
Proposed model	**96.16**	**93.27**	**94.55**	**0.9762**	**0.9111**

Bold values are for the best performing model.

**Table 6:** Comparison of the proposed model with the previous methods in the AMP-Combined dataset.

Classifier	Accuracy (%)	Precision (%)	Recall (%)	ROC-AUC	MCC
AMPDeep^19^	86	**90.91**	80	0.8964	0.7252
Proposed model	**89.28**	87.59	**86.41**	**0.942**	**0.7788**

Bold values are for the best performing model.

Table 7 presents the performance metrics of the model without the CNNs module across three datasets: RNN-Hem, Hlppredfuse, and AMP-Combined. This table is crucial for understanding the impact of the CNNs module on the overall model performance and identifying its supportive contribution. In the RNN-Hem dataset, removing the CNNs module led to a decrease in accuracy from 79.69 to 74.02%, precision from 82.93 to 79.04%, recall from 76.69 to 68.05%, ROC-AUC from 0.861 to 0.7424, and MCC from 0.5962 to 0.4877. Similarly, in the Hlppredfuse dataset, the model without CNNs showed reduced performance in accuracy, precision, recall, ROC-AUC, and MCC compared to the full model. The AMP-Combined dataset also exhibited lower metrics without the CNNs module, indicating its significant contribution to the model's predictive capabilities across different datasets.

**Table 7:** Performance of the model without CNN in the three data sets.

Dataset	Accuracy (%)	Precision (%)	Recall (%)	ROC-AUC	MCC
RNN-Hem^18^	74.02	79.04	68.05	0.7424	0.4877
Hlppred-fuse^12^	94.89	94.23	89.09	0.9331	0.8799
AMP-Combined^19^	86.98	86.32	81.54	0.8619	0.7305

**Figure 3:**
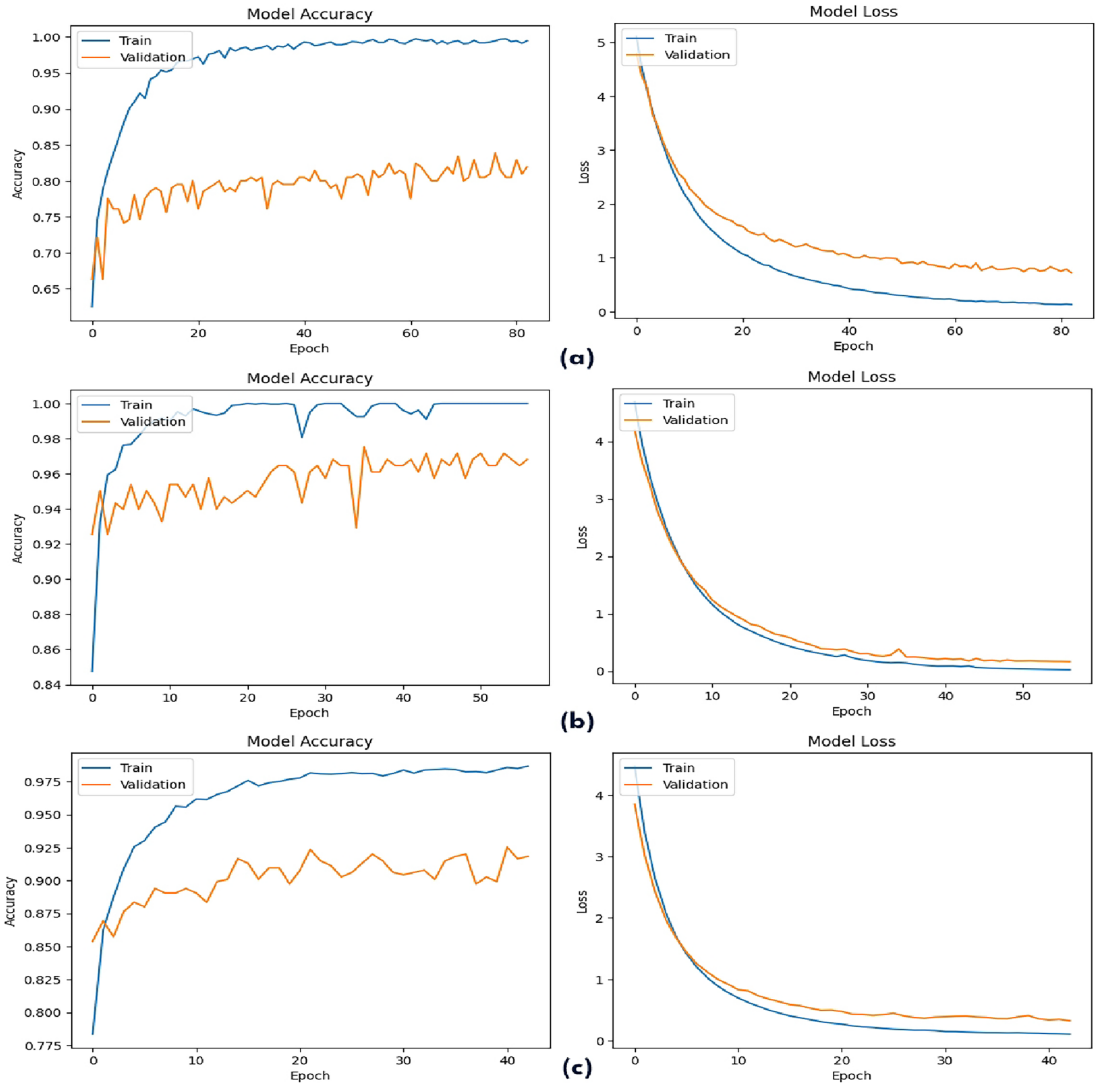
Model accuracy and loss curve for three datasets (**a**) RNN-Hem, (**b**) Hlppredfuse, and (**c**) Combined.

To examine the model learning process, we visualized its accuracy and loss curves in the three data sets, as shown in Fig. 3. In particular, the accuracy curves for all datasets exhibited a consistent upward trend, indicating successful learning and convergence towards optimal performance. This pattern was particularly evident for the Hlppredfuse dataset, where the model achieved remarkable accuracy during training. Loss curves showed a steady downward trajectory, reflecting a gradual reduction in prediction errors as training progressed. This decline was particularly pronounced for the AMP-Combined dataset, demonstrating efficient error minimization. Collectively, these curves affirm the model's ability to effectively learn from the training data and refine its predictive capabilities over time. This robust learning behavior underpins the model's exceptional performance in predicting peptide hemolytic activity. This decline was particularly pronounced for the Combined dataset, demonstrating efficient error minimization. Collectively, these curves affirm the model's ability to effectively learn from the training data and refine its predictive capabilities over time. This robust learning behavior underpins the model's exceptional performance in predicting peptide hemolytic activity.

The training process is a critical aspect of model development and influences both the time required for convergence and the complexity of the trained model. Table 8 provides information on the training time for each dataset and the corresponding number of trainable parameters in the proposed hybrid transformer-CNN model. The proposed model comprises a total of 11,748,097 trainable parameters, indicating the complexity of the neural network architecture. This parameter count encompasses the weights and biases in the convolutional and transformer layers, as well as the fully connected layers, contributing to the model's ability to capture intricate patterns within peptide sequences.

**Table 8:** Training time and training parameters that were associated with the proposed model.

Dataset	Training time (seconds)	Trainable parameters
RNN-Hem^21^	52.78	11,748,097
Hlppredfuse^12^	50.25	11,748,097
Combined^19^	102.55	11,748,097

## Conclusions

In conclusion, our research presents an innovative computational method for forecasting the hemolytic potential of peptides. By combining the strengths of CNNs and transformer-based attention mechanisms, our hybrid transformer-CNN model can detect complex patterns within peptide sequences. This results in highly accurate predictions of hemolytic activity. Our model's success can be seen in its performance on various datasets, such as RNN-Hem, Hlppredfuse, and Combined. The proposed method achieved the highest prediction accuracy with Matthews’s correlation coefficients of 0.5962, 0.9111, and 0.7788 on these datasets, respectively. Comparative analyses highlight the competitive or superior performance of our hybrid Transformer-CNN architecture model compared to existing classifiers. Across the RNN-Hem, Hlppredfuse, and Combined datasets, our model outperforms or matches the performance of established methods, demonstrating its effectiveness in addressing the challenges associated with predicting hemolytic potential. Despite these successes, our model has limitations that must be considered. The model's performance is heavily dependent on the quality and diversity of the training datasets. The current datasets may not cover all possible peptide variations, potentially affecting the model's generalizability. The computational intensity required for training and optimizing the model may not be accessible to all researchers, given the need for high-performance GPUs and substantial memory capacity. The complexity of the model poses challenges in interpretability, the predictions generated by the model need to be experimentally validated to confirm their accuracy and reliability in real-world scenarios. Future research could explore the extension of our model to additional datasets, further validating its generalizability. Additionally, fine-tuning the model's hyperparameters and exploring different architectural configurations may offer opportunities for refinement and improvement. Our work sets the stage for continued advancements in predictive modeling of hemolytic activity, with potential implications for the broader fields of bioinformatics and drug discovery. Finally, partially ordered sets can be used according to their effect on red blood cell hemolysis, presenting a promising direction for future investigations.

## Data Availability

The data and the scripts for this work are available through GitHub at https://github.com/mohamedelhakim/Transformer-CNN-Architecture and You can run the code on the link: https://www.kaggle.com/code/mohamedelhakim/cnn-trens-paper.

## References

[1] Hancock REW, Sahl H-G (2006). Antimicrobial and host-defense peptides as new anti-infective therapeutic strategies. Nat. Biotechnol..

[2] Gostaviceanu A, Gavrilaş S, Copolovici L, Copolovici DM (2023). Membrane-active peptides and their potential biomedical application. Pharmaceutics.

[3] Carpenter AM, van Hoek ML (2024). Development of a defibrinated human blood hemolysis assay for rapid testing of hemolytic activity compared to computational prediction. J. Immunol. Methods.

[4] Oddo A, Hansen PR (2017). Hemolytic activity of antimicrobial peptides. Methods Mol. Biol. Clifton NJ.

[5] Zhao J, Zhao C, Liang G, Zhang M, Zheng J (2013). Engineering antimicrobial peptides with improved antimicrobial and hemolytic activities. J. Chem. Inf. Model..

[6] Indolia S, Goswami AK, Mishra SP, Asopa P (2018). Conceptual understanding of convolutional neural network—A deep learning approach. Procedia Comput. Sci..

[7] Chandra A, Tünnermann L, Löfstedt T, Gratz R (2023). Transformer-based deep learning for predicting protein properties in the life sciences. eLife.

[8] Robles-Loaiza AA (2022). Traditional and computational screening of non-toxic peptides and approaches to improving selectivity. Pharm. Basel Switz..

[9] Wu X (2014). In vitro and in vivo activities of antimicrobial peptides developed using an amino acid-based activity prediction method. Antimicrob. Agents Chemother..

[10] Yaseen A, Gull S, Akhtar N, Amin I, Minhas F (2021). HemoNet: Predicting hemolytic activity of peptides with integrated feature learning. J. Bioinform. Comput. Biol..

[11] Nambiar, A. et al. Transforming the language of life: Transformer neural networks for protein prediction tasks. In *Proceedings of the 11th ACM International Conference on Bioinformatics, Computational Biology and Health Informatics* 1–8 (Association for Computing Machinery, New York, NY, USA, 2020). 10.1145/3388440.3412467.

[12] Hasan MM (2020). HLPpred-Fuse: improved and robust prediction of hemolytic peptide and its activity by fusing multiple feature representation. Bioinforma. Oxf. Engl..

[13] Plisson F, Ramírez-Sánchez O, Martínez-Hernández C (2020). Machine learning-guided discovery and design of non-hemolytic peptides. Sci. Rep..

[14] Timmons PB, Hewage CM (2020). HAPPENN is a novel tool for hemolytic activity prediction for therapeutic peptides which employs neural networks. Sci. Rep..

[15] Wang G, Vaisman II, van Hoek ML (2022). Machine learning prediction of antimicrobial peptides. Methods Mol. Biol. Clifton NJ.

[16] Chaudhary K (2016). A web server and mobile app for computing hemolytic potency of peptides. Sci. Rep..

[17] Rengifo-Lema MJ, Proaño-Bolaños C, Cuesta S, Meneses L (2024). Computational modelling of the antimicrobial peptides Cruzioseptin-4 extracted from the frog *Cruziohyla calcarifer* and Pictuseptin-1 extracted from the frog *Boana picturata*. Sci. Rep..

[18] Capecchi A (2021). Machine learning designs non-hemolytic antimicrobial peptides. Chem. Sci..

[19] Salem M, Keshavarzi Arshadi A, Yuan JS (2022). AMPDeep: Hemolytic activity prediction of antimicrobial peptides using transfer learning. BMC Bioinform..

[20] Birnbaum, S., Kuleshov, V., Enam, Z., Koh, P. W., Ermon, S. Temporal FiLM: Capturing long-range sequence dependencies with feature-wise modulations. Preprint at 10.48550/arXiv.1909.06628 (2021).

[21] Dollar, P., Tu, Z., Tao, H. & Belongie, S. Feature Mining for Image Classification. In *2007 IEEE Conference on Computer Vision and Pattern Recognition* 1–8. 10.1109/CVPR.2007.383046 (2007).

[22] Jin J, Xu F, Liu Z, Shuai J, Li X (2024). Quantifying the underlying landscape, entropy production and biological path of the cell fate decision between apoptosis and pyroptosis. Chaos Solitons Fractals.

[23] Jin J (2023). Biphasic amplitude oscillator characterized by distinct dynamics of trough and crest. Phys. Rev. E.

[24] Li X (2021). RIP1-dependent linear and nonlinear recruitments of caspase-8 and RIP3 respectively to necrosome specify distinct cell death outcomes. Protein Cell.

[25] DiMaggio PA, Floudas CA, Lu B, Yates JR (2008). A hybrid method for peptide identification using integer linear optimization, local database search, and quadrupole time-of-flight or OrbiTrap tandem mass spectrometry. J. Proteome Res..

[26] Badr E, Selim IM, Mostafa H, Attiya H (2022). An integer linear programming model for partially ordered sets. J. Math..

[27] Badr EM, Moussa MI (2020). An upper bound of radio k-coloring problem and its integer linear programming model. Wirel. Netw..

[28] Badr E, El-Hakeem M, El-Sharawy EE, Ahmed TE (2023). An efficient algorithm for decomposition of partially ordered sets. J. Math..

[29] Hu H (2023). Gene function and cell surface protein association analysis based on single-cell multiomics data. Comput. Biol. Med..

[30] Wang W, Zhang L, Sun J, Zhao Q, Shuai J (2022). Predicting the potential human lncRNA-miRNA interactions based on graph convolution network with conditional random field. Brief. Bioinform..

[31] Zhang L, Yang P, Feng H, Zhao Q, Liu H (2021). Using network distance analysis to predict lncRNA-miRNA interactions. Interdiscip. Sci. Comput. Life Sci..

[32] Chen Z (2023). DCAMCP: A deep learning model based on capsule network and attention mechanism for molecular carcinogenicity prediction. J. Cell. Mol. Med..

[33] Meng R, Yin S, Sun J, Hu H, Zhao Q (2023). scAAGA: Single cell data analysis framework using asymmetric autoencoder with gene attention. Comput. Biol. Med..

[34] Zhao J, Sun J, Shuai SC, Zhao Q, Shuai J (2023). Predicting potential interactions between lncRNAs and proteins via combined graph auto-encoder methods. Brief. Bioinform..

[35] Wang J (2024). Predicting drug-induced liver injury using graph attention mechanism and molecular fingerprints. Methods.

[36] Gao H (2023). Predicting metabolite-disease associations based on auto-encoder and non-negative matrix factorization. Brief. Bioinform..

[37] Wang T, Sun J, Zhao Q (2023). Investigating cardiotoxicity related with hERG channel blockers using molecular fingerprints and graph attention mechanism. Comput. Biol. Med..

[38] Sun F, Sun J, Zhao Q (2022). A deep learning method for predicting metabolite-disease associations via graph neural network. Brief. Bioinform..

[39] Abdelbaky I, Tayara H, Chong KT (2022). Identification of miRNA-small molecule associations by continuous feature representation using auto-encoders. Pharmaceutics.

[40] Bebis G, Georgiopoulos M (1994). Feed-forward neural networks. IEEE Potentials.

[41] Chicco D, Tötsch N, Jurman G (2021). The Matthews correlation coefficient (MCC) is more reliable than balanced accuracy, bookmaker informedness, and markedness in two-class confusion matrix evaluation. BioData Min..

[42] Win TS (2017). HemoPred: A web server for predicting the hemolytic activity of peptides. Future Med. Chem..

